# New insights into plant glycoside hydrolase family 32 in Agave species

**DOI:** 10.3389/fpls.2015.00594

**Published:** 2015-08-05

**Authors:** Emmanuel Avila de Dios, Alan D. Gomez Vargas, Maura L. Damián Santos, June Simpson

**Affiliations:** Department of Genetic Engineering, Centro de Investigación y Estudios AvanzadosIrapuato, Mexico

**Keywords:** agave, fructans, plant glycoside hydrolase family 32, amino acid motifs, transcriptome analysis, *in silico* expression, floral tissue

## Abstract

In order to optimize the use of agaves for commercial applications, an understanding of fructan metabolism in these species at the molecular and genetic level is essential. Based on transcriptome data, this report describes the identification and molecular characterization of cDNAs and deduced amino acid sequences for genes encoding fructosyltransferases, invertases and fructan exohydrolases (FEH) (enzymes belonging to plant glycoside hydrolase family 32) from four different agave species (*A. tequilana, A. deserti, A. victoriae-reginae*, and *A. striata*). Conserved amino acid sequences and a hypervariable domain allowed classification of distinct isoforms for each enzyme type. Notably however neither 1-FFT nor 6-SFT encoding cDNAs were identified. *In silico* analysis revealed that distinct isoforms for certain enzymes found in a single species, showed different levels and tissue specific patterns of expression whereas in other cases expression patterns were conserved both within the species and between different species. Relatively high levels of *in silico* expression for specific isoforms of both invertases and fructosyltransferases were observed in floral tissues in comparison to vegetative tissues such as leaves and stems and this pattern was confirmed by Quantitative Real Time PCR using RNA obtained from floral and leaf tissue of *A. tequilana*. Thin layer chromatography confirmed the presence of fructans with degree of polymerization (DP) greater than DP three in both immature buds and fully opened flowers also obtained from *A. tequilana*.

## Introduction

Carbohydrates produced by photosynthesis can be consumed directly or stored in plant cells in the form of sucrose, starch or fructans. Whereas, the majority of plants accumulate starch, around 15% of plant species store carbohydrates in the form of fructans (Hendry, 1987). Long term storage of carbohydrates in the form of fructan polymers has been reported in root and stem tissue in dicotyledonous plants such as chicory (*Cichorium intybus*) (Van den Ende and Van Laere, 1996) and Jerusalem artichoke (*Helianthus tuberosus*) (van der Meer et al., 1998), in non-gramineous monocotyledons such as asparagus (*A. officinalis*) (Cairns, 1992) and agave (*Agave tequilana*) (Mancilla-Margalli and López, 2006) and in gramineous species such as wheat (*Triticum aestivum)* (Housley and Daughtry, 1987) and barley (*Hordeum vulgare*) (Wagner and Wiemken, 1986). It has been proposed that fructan polymers serve not only as energy reserves for new growth in the Spring but also to protect cell membranes and contribute to cold tolerance at low Winter temperatures (Hisano et al., 2008). Hendry (1993) also suggested that fructan metabolism is an evolutionary adaptation to withstand prolonged periods of drought. Perhaps surprisingly, fructans are not found exclusively in leaves or storage organs but also in floral tissue where their primary role may be in mechanisms that lead to flower opening by modulating the osmotic state (Bieleski, 1993; Vergauwen et al., 2000).

Most agave species including *Agave tequilana* are well adapted to grow under arid or semi-arid conditions due to a unique combination of several characteristics. Morphological traits include succulence, concave leaves with thick cuticles organized in a rosette formation and shallow adventitious roots (Gentry, 1982) whereas physiological components include CAM mediated photosynthesis (Nobel, 1976) and fructan accumulation (López et al., 2003). The capacity to produce and store fructans has been exploited in Mexico since the pre-Columbian era to produce fermented (pulque) or distilled (tequila, mezcal) beverages (García-Mendoza, 1992) and Agavins (Agave fructan polymers) are of the neoseries type (López et al., 2003).

Agavin structure implies the activity of four different fructosyltransferase enzyme activities: Sucrose:sucrose 1-fructosyltransferase (1-SST), Fructan:fructan 1-fructosyl transferase (1-FFT), sucrose:fructan 6 fructosyltransferase (6-SFT), and Fructan:Fructan 6G-Fructosyltransferase (6G-FFT). Previously different isoforms of 1-SST and 6G-FFT enzymes from *A. tequilana* (shown in red in Supplementary Table 1) were characterized at the genetic and functional level in different tissues and plants of different ages (Cortés-Romero et al., 2012). A 1-FFT type enzyme has also been reported for *A. tequilana* and *A. inaequidans* and shown to respond differentially in terms of expression in relation to exposure to different metabolites such as hormones or sugars (Suárez-González et al., 2013). The commercial importance of *A. tequilana* as a crop and the increasing interest in the exploitation of agave species for biofuel production (Borland et al., 2009; Cushman et al., 2015) underline the need for further detailed analysis of both the synthetic and degradative components of fructan metabolism in agave at the molecular level, with the aim of increasing the efficiency of fructan production and/or producing specific forms of fructan polymers.

Plant glycoside hydrolase family 32 (PGHF32) that includes fructosyltransferases, invertases, and fructan exohydrolases (FEH) is characterized by highly conserved amino acid sequences where changes in a single residue can modify the activity of specific enzymes (Le Roy et al., 2007; Van den Ende et al., 2009). This has necessitated the heterologous expression of putative PGHF32 encoding genes, protein purification and *in vitro* analysis of activity in order to reliably classify genes encoding each enzyme type. In the current environment of massive accumulation of sequence data from a wide number of species the need for analysis of enzyme activity could hamper detailed analysis of fructan metabolism in processes such as stress tolerance and osmotic balance especially in non-model species such as agave. Although definitive activity analysis is essential, an accurate sequence based method for initial classification of putative PGHF32 members at least for agave species would be a useful tool.

Recently transcriptome data has been generated for four different *Agave* species: *A. deserti* and *A. tequilana* (members of the sub-genus *Agave*) (Gross et al., 2013) and *A. victoriae-reginae* and *A. striata* (members of the sub-genus *Littae)* (Avila de Dios and Simpson unpublished). In this work we describe the identification of previously uncharacterized members of PGHF32 from four different Agave species based on RNAseq data and suggest that the hypervariable loop domain (Van den Ende et al., 2009) could be useful for accurate sequence based prediction of enzyme activity. *In silico* expression patterns and qRT-PCR analysis for both newly identified and previously characterized cDNAs indicated well conserved patterns of expression in the three Agave species analyzed with high levels of expression for genes encoding degradative enzyme types in floral tissue. TLC analysis confirmed the presence of fructan polymers in immature buds and flowers of *A. tequilana*.

## Materials and methods

### Agave transcriptome database searches

In order to uncover new members of PGHF32 in agave species, searches were carried out in transcriptome databases from four *Agave* species: *A. deserti, A. tequilana, A. victoriae-reginae*, and *A. striata*. For *A. deserti* and *A. tequilana* transcriptome data was generated by Ilumina Hi-seq and is available at NCBI (Gross et al., 2013). Transcriptomes for *A. victoriae-reginae, A. striata* and a second *A. tequilana* transcriptome were generated individually at Cinvestav Irapuato by Ilumina My-seq in paired-end runs to produce >23 million reads for each species which were assembled using Trinity (Grabherr et al., 2011) and BLAST2GO (Conesa et al., 2005) was used to obtain biological information about the assembled contigs (Avila de Dios and Simpson, unpublished). BLAST (Altschul et al., 2009) searches were carried out on each individual species database to identify fructosyltransferases, invertases and FEH using previously reported sequences from *A. tequilana* and *T. aestivum* as queries Supplementary Table 1. Sequences with *e* < 10-5 were chosen and the open reading frame (ORF) http://www.bioinformatics.org/sms2/orf_find.html, for each determined using ORF finder. Only sequences encoding an ORF representing the complete, predicted protein sequences were selected for comparison with previously characterized amino acid sequences from agave and other species for confirmation of identity. Accession numbers for all sequences used in alignments are listed in Supplementary Table 1. Newly identified and characterized sequences have been deposited in GenBank under accession numbers KR138447-KR138458.

### Alignment of amino acid sequences and identification of conserved motifs

Translated complete ORFs or selected motifs were aligned with MUSCLE (Edgar, 2004) to sequences from agave and other species, which had been validated experimentally. The best substitution model and phylogenetic reconstruction were carried out by maximum likelihood using MEGA 6 (Tamura et al., 2013) and Bootstrap analysis using 1000 repetitions was also carried out. Analysis of conserved motifs and corresponding figures were accomplished using Geneious® 8.1.3(www.geneious.com).

### *In silico* expression analysis

Expression levels in different agave tissues for transcripts encoding different enzyme types and isoforms were determined *in silico* by mapping sequences, Bowtie 0.12.9 (Langmead, 2010) and RSEM 1.2.0 (Li and Dewey, 2011) to assembled contigs. Expression levels are presented as “transcripts per million” (TPM). Heat maps were created based on the expression data for the transcripts PGHF32 isoforms identified in the three *Agave* species using the heatmap.2 function from the gplots library in the R statistics package version 2.17 http://www.R-project.org/.

### qRT-PCR analysis

RNA extraction and qRT-PCR analysis was carried out as described in Abraham Juárez et al. (2015). Primers used are listed in Cortés-Romero et al. (2012).

### Extraction and thin layer chromatography of fructans

Fructans were extracted from ground, lyophilized tissue from approximately 2.5 cm long unopened flower buds or fully opened flowers. Two aqueous extractions were carried out as follows: 30 ml of distilled water was added to 0.2 g of plant tissue and incubated at 75 ± 5°C for 30 min. The supernatant was recovered and the sample was re-extracted in 20 ml distilled water at 75 ± 5°C for 15 min. Both supernatants were combined and frozen before lyophilization to obtain a white powder. This protocol was adapted from Mellado-Mojica and Lopez (2012). Extracted fructans were resuspended in distilled water to a concentration of 25 mg/ml. One microliter of each fructan sample was applied to an aluminum backed silica-gel plate (Sigma–Aldrich) and run three times using a butanol-glacial acetic acid-water (50:25:25 v/v/v) system (Thome and Kühbauch, 1985). Visualization of separated fructans was carried out using the aniline: diphenylamine: phosphoric acid reagent in acetone (Anderson et al., 2000).

## Results

### Identification of members of PGHF32 in agave species

**Figure 1:**
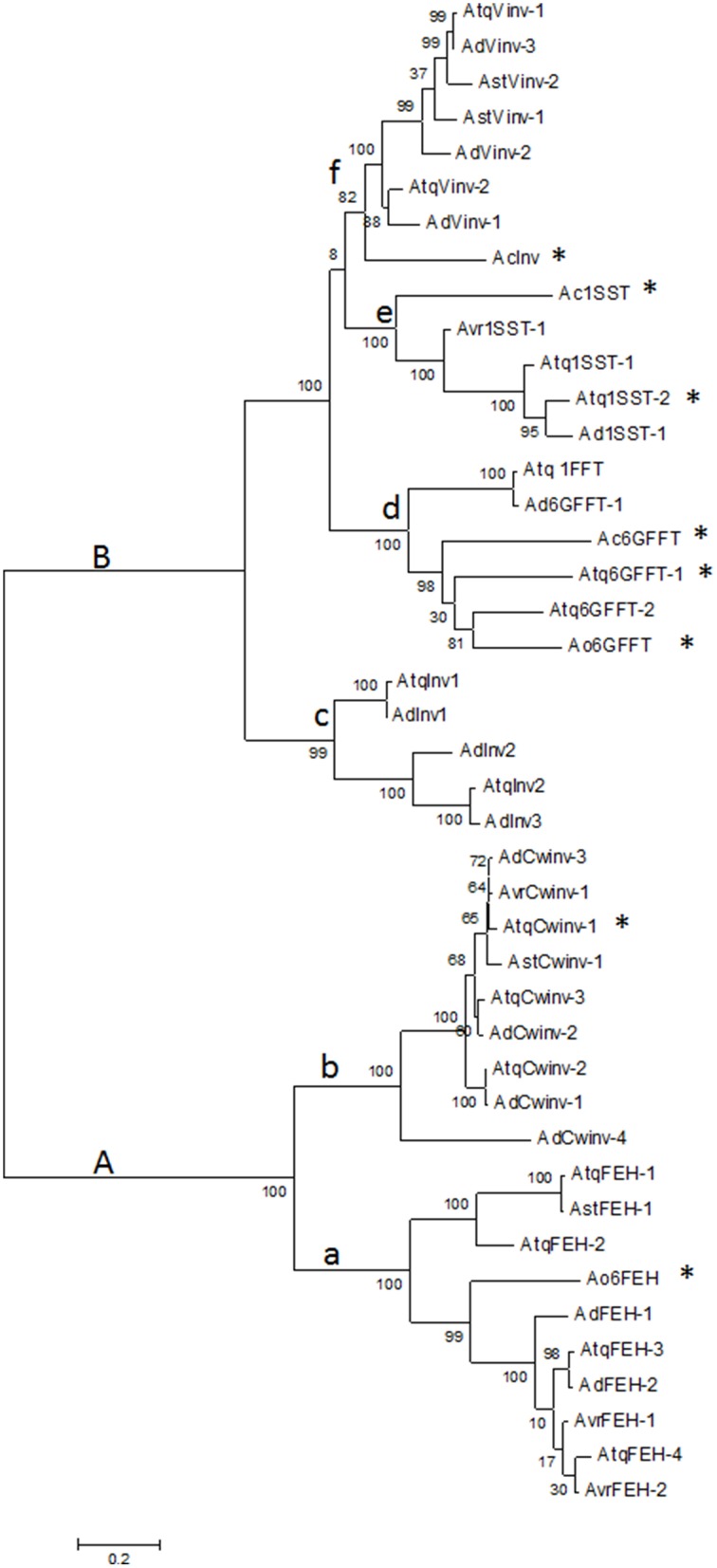
**Dendrogram showing the relationship between amino acid sequences of PGHF32 members from four agave species**. Letters indicate different clades, sequences with activity confirmed by *in vitro* analysis are indicated by ^*^. Atq-*A. tequilana*, Ad-*A. deserti*, Avr- *A. victoriae-reginae*, Ast-*A. striata*, Ao-*A. officinalis*, Ac-*A. cepa*. Numbers indicate Bootstrap values.

Transcriptome databases for four different *Agave* species: *A. tequilana, A. deserti, A. victoriae-reginae*, and *A. striata* were analyzed in order to identify sequences encoding members of PGHF32. In total 255 transcripts encoding putative PGHF32 members were identified and 31 new full-length cDNA sequences determined. The predicted amino acid sequences for each of the full length cDNAs were aligned and compared with previously characterized amino acid sequences (indicated by ^*^ in Figure 1) for 1-SST and 6G-FFT fructosyltransferases, vacuolar (Vinv) and cell wall (Cwinv) invertases and FEH from *A. tequilana, A. officinalis*, and *A. cepa* (closely related members of the Asparagales family) whose activity had been confirmed previously by heterologous expression and *in vitro* assays. As expected from previous reports, two main groups are formed: A containing FEH (subgroup a) and cell wall invertases (subgroup b) and B containing fructosyltransferases (subgroups d-6G-FFT and e-1-SST), vacuolar invertases (subgroup f) and an undefined invertase clade (subgroup c) (Figure 1). Based on these groups each sequence could be tentatively classified as encoding a specific enzyme type and sequences were named based on this classification and the agave species from which they were obtained. Sequences in the same clade from the same species representing putatively different isoforms had at least 4% divergence at the amino acid level. As can be observed sequences putatively encoding fructan exohydrolase enzymes (FEH) were identified for the first time for *A. tequilana* and new isoforms for vacuolar and cell wall type invertases were also determined for this species. Enzymes in each class were identified for *A. deserti* but complete sequences could only be identified for invertase and FEH type enzymes from *A. striata* and invertase, FEH and 1-SST type enzymes for *A. victoriae-reginae*. The numbers and types of new isoforms found for each species are summarized in Table 1. A dendrogram based on an expanded alignment including all available amino acid sequences from experimentally determined members of PGHF32 from dicotyledonous and monocotyledonous species confirmed the classification of the agave sequences (Supplementary Figure 1). Previously characterized sequences from *A. tequilana* (Cortés-Romero et al., 2012) are indicated in red.

**Table 1:** **Summary of newly identified members of PGHF32 in *Agave* species**.

	**1-SST**	**6G-FFT**	**Vinv**	**Cwinv**	**FEH**
*Agave tequilana*	–	–	3	2	4
*Agave deserti*	1	1	6	4	2
*Agave striata*	–	–	2	1	1
*Agave victoriae-reginae*	1	–	–	1	2

Based on this data, members of PGHF32 for *A. victoriae-reginae, A. striata*, and *A. deserti* are reported for the first time. FEH isoforms and novel invertase isoforms were also determined for *A. tequilana* including a putatively distinct invertase isoform found only in *A. tequilana* and *A. deserti*.

### Comparison of conserved motifs and differences within the hypervariable loop domain

**Figure 2:**
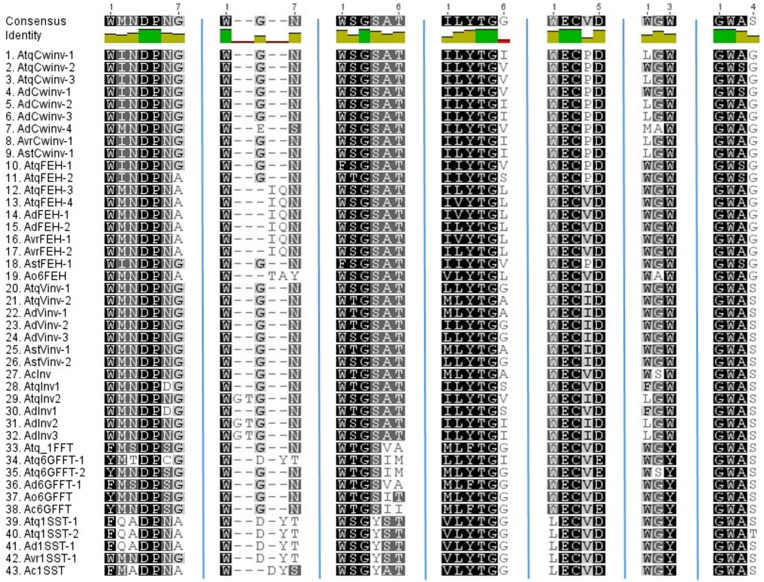
**Comparison of conserved amino acid motifs specific to PGHF32**. Motifs are indicated above each column. Dark green squares indicate residues conserved in all sequences.

The conserved motifs close to the active sight that characterize PGHF32 are shown in Figure 2. The FRDP motif is not displayed since this motif was perfectly conserved in all sequences analyzed. In general the WMNDNPG, WSGSAT, ILYTGG, WECPD (WECVD), and GWAS motifs are well conserved within all PGHF32 members from the four *Agave* species analyzed. Differences observed previously for the WMNDNPG and WSGSAT motifs in fructosyltransferases of *A. tequilana* were confirmed and also shown to be present in other agave species. The undefined invertase group (clade c in Figure 1) shows no specific pattern of amino acid conservation for these motifs.

The hypervariable loop region has previously been shown to contain conserved arrangements of amino acids that correlate with enzyme type. When an 18 amino acid sequence spanning this region was used to compare the members of PGHF32 from *Agave* species, a strong correlation between conserved amino acids and enzyme activity was observed (Supplementary Figure 2). In order to determine whether the 18 amino acid region could be useful as a general tool for distinguishing and identifying the different enzyme types within PGHF32, a comparison was made of all available complete amino acid sequences from both monocotyledonous and dicotyledonous species, encoding enzymes whose activity had been experimentally confirmed (Supplementary Figure 3).

**Figure 3:**
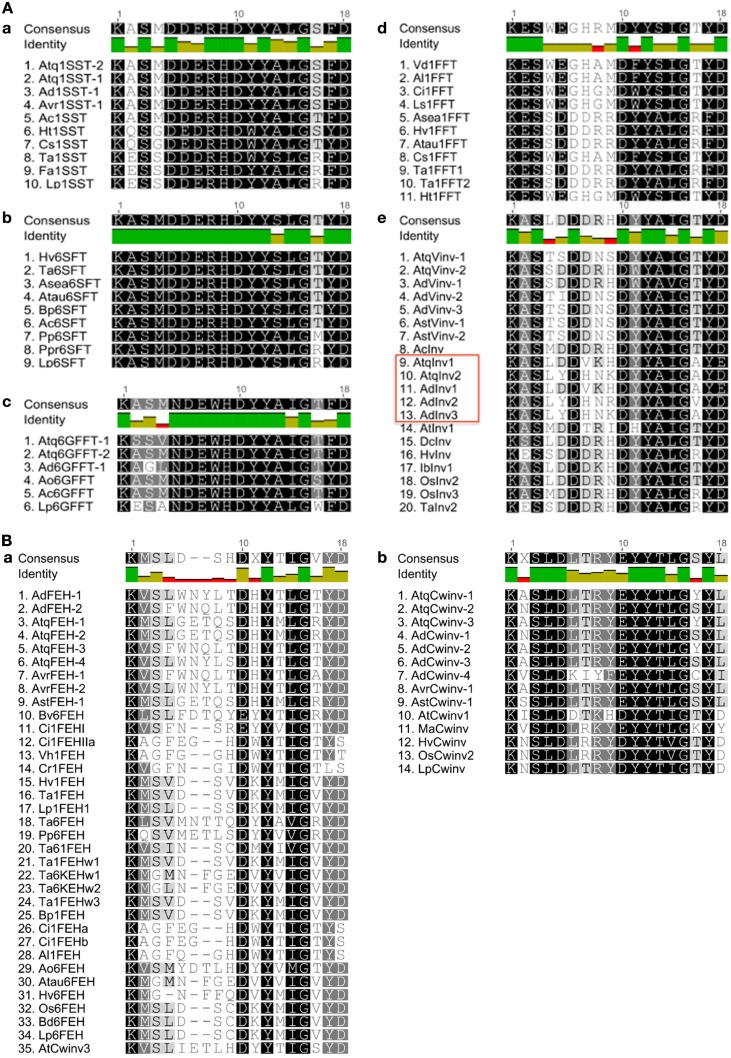
**Comparison of amino acid sequences within the 18 amino acid hypervariable region**. **(A)** Sequences from fructosyltransferases (a–d) and vacuolar invertases (e). The red box indicates a putatively new invertase isoform. **(B)** Sequences from (a) fructan exohydrolases (AtCwinv3 has been re classified as an FEH) and Cell wall invertases (b). Dark green squares indicate residues conserved in all sequences.

Based only on the 18 amino acid hypervariable loop region a reasonably good correlation is obtained between the groups formed in the dendrogram and enzyme activity. Only 5 of the 106 sequences indicated with red boxes in Supplementary Figure 3 show no strong correlation between activity and the groups defined by sequence analysis. AtCwinv3 indicated by a stippled red box was initially classified as a cell wall invertase but later shown to be an FEH and is correctly placed in the corresponding clade. When the 18 amino acid loop sequences were aligned by putative or confirmed enzyme activity, distinct patterns of conserved amino acids could be determined for each of the enzyme types (Figures 3A,B). As indicated, three amino acids: lysine, tyrosine, and glycine at positions 1, 12, and 15 respectively within the 18 amino acid motif are conserved in all sequences. This minimal pattern distinguishes the FEH group from the other enzyme types. In contrast the closely related Cwinv group, in addition to the minimal 3, has 6 additional conserved amino acids, the vacuolar invertases 5 and the 1-SST, 6G-FFT, and 1FFT fructosyltransferases 6, 9, and 5 additional conserved amino acids respectively (Table 2). The conserved amino acids within the 18-residue motif for the 6SFT type enzyme is based on closely related reported sequences whose identities have not yet been confirmed by activity and shows strong conservation with only 2 of the 18 residues found to vary. Although Cwinvs and FEHs could clearly be distinguished based on the hypervariable loop region, different forms of FEH type enzymes could not be accurately determined. The newly identified Vinv, Cwinv, FEH, and FFT genes from the different agave species show good correlations with the conserved amino acid patterns and putative enzyme types, supporting the initial classification based on the dendrogram in Figure 1.

**Table 2:** **Summary of conserved amino acids in the hypervariable loop domain**.

	**Conserved amino acids in hypervariable loop region**																	
Enzyme Type	1	2	3	4	5	6	7	8	9	10	11	12	13	14	15	16	17	18
FEH	K	–	–	–	–	–	–	–	–	–	–	Y	–	–	G	–	–	–
Cwinv	K	–	S	L	D	–	–	–	–	–	Y	Y	T	–	G	–	Y	–
Vinv	K	–	S	–	–	D	–	–	–	D	–	Y	A	–	G	–	Y	–
6G-FFT	K	–	–	–	N	D	E	W	H	D	Y	Y	A	–	G	–	–	D
1-SST	K	–	S	–	D	–	–	R	H	D	–	Y	–	–	G	–	–	D
1-FFT	K	E	S	–	E	–	–	–	–	D	–	Y	–	–	G	–	–	D
6-SFT	K	A	S	M	D	D	E	R	H	D	Y	Y	–	L	G	–	Y	D

The undefined group (clade c in Figure 1) boxed in e of Figure 3A, shows the same pattern of conserved amino acids as the Vinvs supporting their classification as invertases. However, an alignment of the complete amino acid sequences for clades c and f from Figure 1 uncovered a sequence structure specific to clade c where in addition to conserved amino acid sequences, defined groups of amino acids are either missing or inserted in comparison with clade f (red boxes, Supplementary Figure 4) and may indicate functional differences.

Highly conserved motifs confirm the identification of new members of PGHF32 in agave species and specific arrangements of conserved amino acids within the hypervariable loop domain could be exploited at least for preliminary classification of new sequence data pertaining to PGHF32. No agave sequences were classified as encoding either 1-FFT or 6-SFT type fructosyltransferases.

### *In silico* expression analysis of PGHF32 members in different tissues

**Figure 4:**
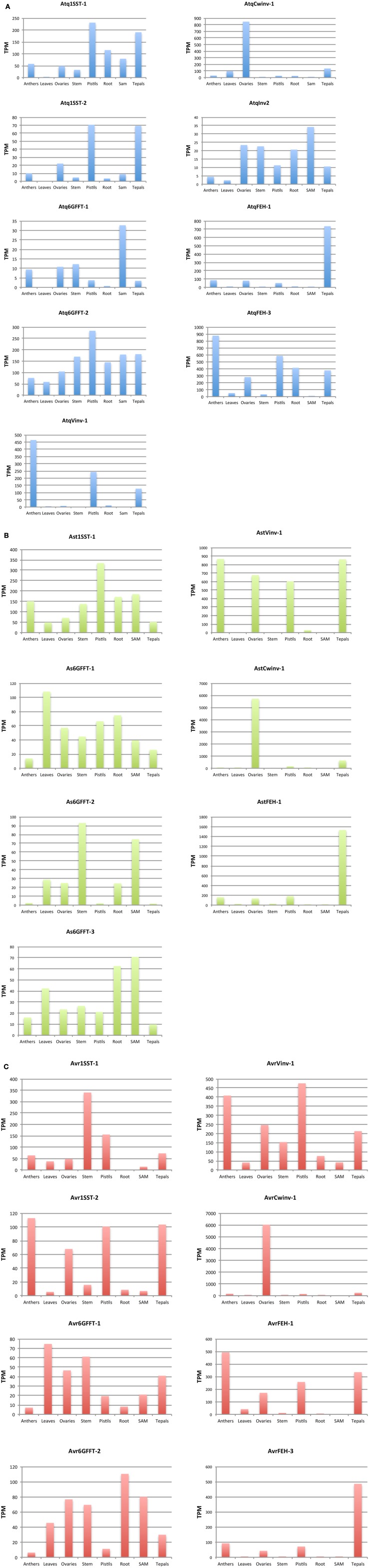
*****In silico*** expression analysis of PGHF32 isoforms from three ***Agave*** species. (A)** Isoforms of *A. tequilana*, **(B)** Isoforms of *A. striata*, **(C)** Isoforms of *A. victoriae-reginae*. TPM-Transcripts per million reads, Enzyme names defined in abreviations.

General transcriptome analysis (data not shown) of PGHF32 genes in *A. tequilana, A. striata*, and *A. victoriae-reginae* revealed expression in tissues such as roots and flowers. In order to document the expression patterns of PGHF32 genes in different tissues of different *Agave* species, *in silico* expression analysis based on the transcriptome databases generated at Cinvestav was carried out for each enzyme type for *A. tequilana, A. striata*, and *A. victoriae-reginae* (Figures 4A–C). Although full-length amino acid sequences were not be identified for all enzyme types in all *Agave* species, mapping of partial transcripts to contigs as described in Materials and Methods, allowed the expression patterns of genes encoding the different enzymes of PGHF32 to be determined, including patterns of expression of different isoforms for specific enzymes within a single species.

### *In silico* expression analysis of PGHF32 members in different tissues

General transcriptome analysis (data not shown) of PGHF32 genes in *A. tequilana, A. striata*, and *A. victoriae-reginae* revealed expression in tissues such as roots and flowers. In order to document the expression patterns of PGHF32 genes in different tissues of different *Agave* species, *in silico* expression analysis based on the transcriptome databases generated at Cinvestav was carried out for each enzyme type for *A. tequilana, A. striata*, and *A. victoriae-reginae* (Figures 4A–C). Although full-length amino acid sequences were not be identified for all enzyme types in all *Agave* species, mapping of partial transcripts to contigs as described in Materials and Methods, allowed the expression patterns of genes encoding the different enzymes of PGHF32 to be determined, including patterns of expression of different isoforms for specific enzymes within a single species.

For *A. tequilana*, as previously reported very similar patterns of expression were observed for *Atq1-SST-1* and *Atq1-SST-2* with highest expression levels in tepals and pistils. In contrast to the *1-SST* isoforms, *Atq6G-FFT-1* and *Atq6G-FFT-2* isoforms show distinct patterns of expression. Whereas, *Atq6G-FFT-2* is expressed in all tissue types and at the highest level in pistils and shows a similar pattern of expression to the *Atq1-SST-1* and *Atq1-SST-2* isoforms, *Atq6G-FFT-1* shows highest expression in shoot apical meristem (SAM) tissue. The newly described *AtqInv2*, also shows highest expression in SAM tissue and overall shows a similar expression pattern to *Atq6G-FFT-1*. With the exception of *AtqInv2* the *A. tequilana* genes encoding invertases or FEHs are expressed to higher levels in comparison to the genes encoding fructosyltransferases and also show unique patterns of expression with each isoform highly expressed in a specific floral tissue Figure 4A. These results are also represented as heat maps in Supplementary Figures 5A,B where Figure S5A is normalized in relation to the isoforms and Figure S5B is normalized in relation to plant tissues. The dendrogram relating to the isoforms (left hand side of figure) in Figure S5A also reflects the similarities in expression patterns between the fructosyltransferase isoforms and the *AtqVinv-2* isoform and the unique expression patterns for each of the other invertase and FEH isoforms.

The single *Ast1-SST-1* isoform shows a similar pattern of expression as the *A. tequilana 1-SST* transcripts with highest expression in pistils. Interestingly three distinct isoforms encoding *6G-FFT* type enzymes (*Ast6G-FFT-1, Ast6G-FFT-2*, and *Ast6G-FFT-3*) were identified for *A. striata* and although all three isoforms are most highly expressed in vegetative tissue such as leaves, stem, SAM and roots, each shows a slightly different expression pattern. These isoforms show similar patterns of expression to *Atq6G-FFT-1* and *AtqInv2*. As observed for *A. tequilana*, the expression patterns observed for the *A. striata* invertase and FEH isoforms are also more highly expressed in comparison to the fructosyltranserase isoforms and also show unique tissue specific patterns Figure 4B. These patterns are reflected in Supplementary Figures 5B,C where the dendrogram relating to the isoforms (left hand side of Figure S5B) show a closely related cluster containing the FT isoforms whereas the Invertase and FEH isoforms are on more distant branches.

The two isoforms of *1-SST* identified for *A. victoriae-reginae* show very different expression patterns. *Avr1-SST-1* is most strongly expressed in stem tissue whereas *Avr1-SST-2* shows highest expression in floral organs corresponding more closely to the expression patterns observed for *Atq1-SST-1, Atq1-SST-2*, and *Ast1-SST-1*. *Avr1-SST-1* shows an expression pattern similar to *Avr6G-FFT1* and *2* that are also most highly expressed in vegetative tissues and to a lower level in floral tissues as was described for *Ast6G-FFT-1, Ast6G-FFT-2*, and *Ast6G-FFT-3* Figure 4C. *AvrVinv-1, AvrCwinv-1, AvrFEH-1*, and *AvrFEH-3* all show expression patterns very similar to the putatively orthologous sequences in the other Agave species as described above. Supplementary Figures 5D,E show these relationships.

*Avr1-SST-1* and *2, Avr6G-FFT-1* and *2, Ast1-SST-1* and *Ast6G-FFT-1, 2*, and *3* are all tentatively named isoforms based on partial sequence comparisons. Transcripts for other isoforms encoding a *1-SST, Cwinvs, Invs*, and *FEHs* were also identified but showed the same expression patterns as those presented in Figure 4 and for brevity have not been included.

In most cases distinct isoforms from a single species showed different levels and tissue specific patterns of expression supporting their classification. Certain FT isoforms showed higher expression in floral tissues whereas others were most highly expressed in vegetative tissues. In general, higher levels and unique patterns of expression for invertases and FEHs were observed in comparison to FTs for all three species in floral tissues in comparison to vegetative tissues.

### Confirmation of expression of selected genes and presence of fructans in floral tissue

**Figure 5:**
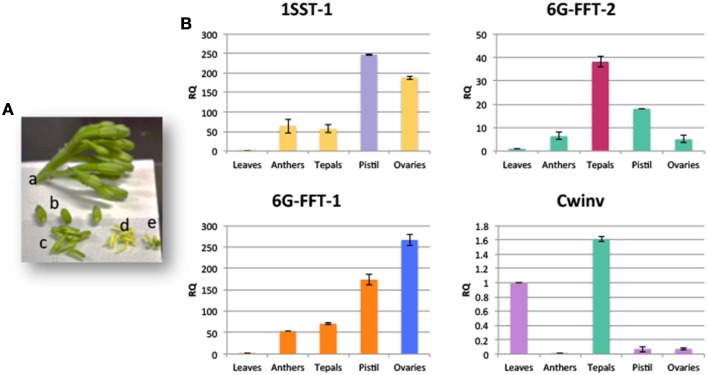
**qRT-PCR analysis of transcripts encoding 1-SST, 6G-FFT, and Cwinv type enzymes in different floral tissues of ***A. tequilana*****. **(A)** Example of flower buds and floral tissues used for qRT-PCR analysis a, complete umbel; b, whole buds; c, dissected tepals; d, anthers; e, pistils and ovaries. **(B)** qRT-PCR expression patterns of PGHF32 genes in floral tissues.

High levels of expression of PGHF32 members in floral tissue have not been documented previously for any Agave species. The *in silico* expression data however show that many of the isoforms identified for Agave PGHF32 enzymes are highly expressed in floral tissue in all three species. In order to confirm these observations, qRT-PCR analysis was carried out for *Atq1-SST-1, Atq6G-FFT-1, Atq6G-FFT-2*, and *AtqCwinv-1* in immature floral buds (Figure 5A) of *A. tequilana* samples not used to obtain transcriptome data. As shown in Figure 5B, the fructosyltransferase encoding genes show higher expression in at least one of the floral tissue types in relation to leaf tissue supporting the results from the *in silico* data. *AtqCwinv-1* was expressed at a low level and no significant difference was observed between floral and leaf tissue in this experiment.

**Figure 6:**
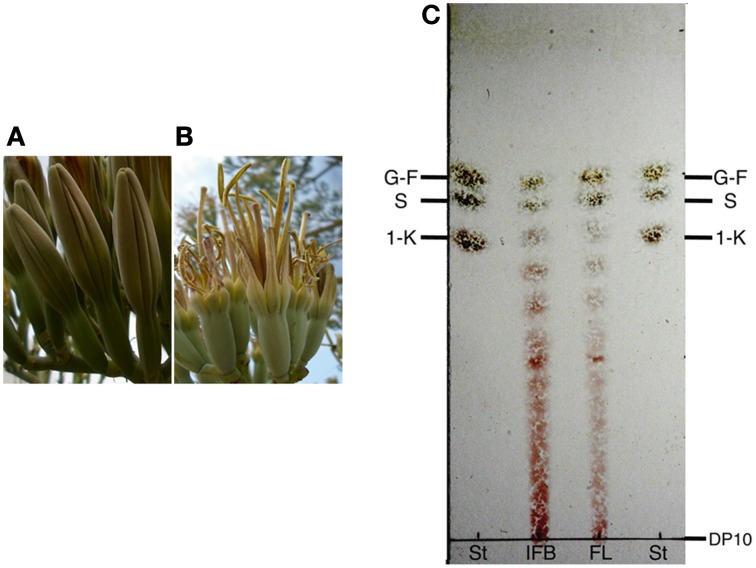
**TLC analysis of floral tissue from ***A. tequilana*****. **(A)** Example of immature floral buds, **(B)** Example of a fully developed *A. tequilana* flower. **(C)** TLC of fructans extracted from: IFB-immature floral buds and FL fully developed *A. tequilana* flowers. G-glucose, F-fructose, S-sucrose, 1-K-1kestose, DP-degree of polymerization. St-standards.

High levels of expression of genes encoding enzymes responsible for fructan synthesis suggest the presence of fructan polymers in *Agave* flowers and in order to confirm this hypothesis, TLC was carried out on extracts obtained from immature flower buds and fully opened *A. tequilana* flowers (Figures 6A,B). As shown in Figure 6C, fructans of between 3° and 10° of polymerization (DP) were observed in immature buds and mature floral tissue although the larger DP fractions are somewhat less abundant in mature floral tissue.

Quantitative real time PCR analysis confirmed high levels of expression of genes encoding PGHF32 enzymes in floral tissue and TLC analysis also showed the presence of fructooligosaccharides (FOS) in these tissues.

## Discussion

The association of the groups obtained in the dendrogram in Figure 1, with sequences from at least one enzyme whose activity had been confirmed allowed the putative allocation of the newly identified sequences into different fructosyltransferase, fructan exohydrolase, and invertase groups and comparison of the agave amino acid sequences with a wider range of more distantly related species including both monocotyledons and dicotyledons also supports the classification of the agave sequences as does the analysis of the variable loop region where each enzyme type, showed the conserved amino acid configurations. Additionally, the presence of closely related sequences and the conservation of expression patterns for isoforms found in different agave species within the same groups also lends weight to the classification.

Previously Cortés-Romero et al. (2012) described 1-SST, 6G-FFT, cell wall and vacuolar invertase encoding sequences for *A. tequilana*. Here we report a new vacuolar invertase isoform, a novel tentatively classified invertase isoform, two new isoforms encoding a cell wall invertase and for the first time four isoforms encoding an FEH type enzyme for this species. All enzyme types are also reported for the first time for *A. deserti*. The identification of a novel invertase type enzyme is supported by the presence of transcripts in both *A. tequilana* and *A. deserti* and the difference in expression pattern observed in comparison to the Vinv isoforms from the other species. The significant differences in amino acid sequence observed for the new invertase group could be due to in part to differential transcript processing and this clade could be specific to agave species found in the sub-genus agave such as *A. tequilana* and *A. deserti* since no equivalent sequences were found in the *A. victoriae-reginae* or *A. striata* transcriptomes. It will be of interest to determine the precise activity of enzymes in this group by *in vitro* analysis. The identification of new Agave isoforms for Cwinvs, Vinvs, and FEHs is consistent with the presence of multiple isoforms for these enzymes in other species. *A. thaliana* and Rice have 9 and 6 Cwinv isoforms, respectively, and both have two Vinv isoforms (Sherson et al., 2003; Ji et al., 2005). Three FEH isoforms have also been described previously for Chicory (*C. intybus*) (Van den Ende et al., 2001) and two for wheat (*T. aestivum*) (Van Den Ende et al., 2003).

Suárez-González et al. (2013) reported a 1-FFT type enzyme for *A. tequilana*, however enzyme activity has not been reported for this gene. Based on the grouping in the dendrogram and the sequence found in the hypervariable loop domain it is possible that this sequence may encode a 6G-FFT type enzyme. Although complete amino acid sequences for 1-SST and 6G-FFT could not be assembled for *A. victoriae-reginae* and *A. striata*, at the nucleotide level partial transcripts encoding these enzymes could be mapped to contigs and used in the *in silico* expression analysis. One interesting observation is that in all the transcriptome analysis that we have carried out to date we have never uncovered sequences that can be convincingly classified as encoding 1-FFT or 6-SFT type enzymes. This is surprising since reports of the biochemical structure of Agave fructans (López et al., 2003) in *A. tequilana* indicate that probably both 6-SFT and 1-FFT enzyme activity is necessary in order to produce these polymers. Ritsema et al. (2003) have previously questioned the need for a separate 1-FFT enzyme in onion (*A. cepa*) a relative of the Agavaceae within the order Asparagales. It is possible that *1-FFT* type genes in *Agave* species are expressed at very low levels or in specific tissues that have not been sampled, or that at least one of the several 6G-FFT isoforms could carry out this activity as has been reported in other species such as *L. perenne* (Lasseur et al., 2006) Asparagus (*A. officinalis*) (Ueno et al., 2005) and onion (*A. cepa*) (Ritsema et al., 2003). The lack of candidate cDNAs encoding 6-SFT type enzymes in the four Agave species studied is also intriguing. This may also be due to low levels of expression or tissue specific expression patterns as suggested for 1-FFT. The presence of a 6-SFT enzyme in *A. cepa* has been reported (Fujishima et al., 2005) but not for *A. officinalis*, two species closely related to the Agavaceae.

The difficulty in distinguishing enzyme type within PGHF32 has been commented before (Van den Ende et al., 2009) and in some cases classification based solely on sequence data has proved erroneous as shown by the *A. thaliana* gene classified as *AtCwinv3* and later shown to have FEH activity (De Coninck et al., 2005). Ultimately the final classification of new genes should be based on activity, however the conserved amino acid patterns within the hypervariable loop domain could provide a simple and relatively accurate initial tool to identify and annotate sequence data for PGHF32. This is ever more important as large scale sequencing projects become more numerous and the possibility to confirm activity for many of the species and sequences analyzed will be impractical. Detailed analysis of the hypervariable domain by site directed mutagenesis could also lead to insights on the activity and the determination of specificity for these enzymes.

Expression patterns for different isoforms encoding degradative enzymes (invertases and fructan exohydrolases) of PGHF32 were completely conserved across all species and strongly correlated to floral organs. This observation agrees with the hypothesis that breakdown of fructan polymers in floral tissue is needed to provide both energy and the osmotic variations thought to play a role in the opening of flowers in other species (Bieleski, 1993; Vergauwen et al., 2000). The expression patterns of genes encoding fructosyltransferase enzymes were more variable with distinct patterns of expression observed for each species. *Atq1-SST-1* and *2, Ast1-SST-1* showed similar expression patterns with highest expression in pistils. For *Avr1-SST-1* and *Avr1-SST-2* very different patterns of expression were observed. Whereas, *Avr1-SST-1* was most strongly expressed in stems, *Avr1-SST-2* showed high levels of expression in all floral tissues. *Atq6G-FFT*-1 shows highest expression in SAM tissue and significant expression in stem tissue but low levels in leaf and root. This gene is also moderately expressed in all floral tissues. In contrast, *Atq6G-FFT*-2 is strongly expressed in all tissues with highest expression in pistils. *6G-FFT* encoding genes from *A. victoriae-reginae* or *A. striata* were most strongly expressed in predominantly vegetative tissues. The differences in expression patterns for the different fructosyltransferase encoding genes may reflect differences in the accumulation or turnover of fructans in flowers from the different species and also the different morphology of the inflorescences defining each subgenus. *A. tequilana* is classified in subgenus *Agave* and has a large paniculate inflorescence while *A. victoriae-reginae* and *A. striata* are classified in subgenus *Littae* with simple spicate inflorescences. It may be possible that low DP fructans can be transported more easily to the spicate flowers directly from the inflorescence where they can be utilized immediately, whereas transport in the paniculate inflorescence may be less efficient given the large numbers of branched umbels, leading to the need to synthesize and store at least short DP fructan polymers in floral tissue until needed and hence the need for 6G-FFT activity.

Quantitative RT-PCR analysis of floral tissue confirmed the higher levels of expression observed for *Atq1-SST-1* and *Atq6G-FFT-1* and *2* in floral tissues in comparison to leaves. *AtqCwinv-1* showed only slightly higher expression in tepals in comparison to leaves in contrast to the pattern observed *in silico* where highest expression was observed almost exclusively in ovaries. This may be due to the different developmental stages at which the flower buds were sampled since qRT-PCR analysis was carried out on immature buds whereas transcriptome data was obtained from fully developed flowers. Given the conservation in amino acid sequences between different isoforms encoding enzymes with the same activity it will be of great interest to obtain genomic sequences in order to study the regulatory basis of the differential expression patterns observed.

Based on the expression data, it was expected that fructan polymers would be detected in floral tissue of agave plants and this was confirmed by TLC analysis of samples from *A. tequilana*. Higher levels of polymerization of fructans in immature buds may reflect that during flower development fructans accumulate but are then degraded to provide an energy source and/or osmotic change, leading to the opening of the fully developed flower as has been proposed for other fructan producing species (van Doorn and Van Meeteren, 2003).

Transcriptome analysis allowed us to identify for the first time, cDNAs encoding members PGHF32 in *A. victoriae-reginae, A. striata*, and *A. deserti* and to obtain sequences to complete the set of enzymes necessary to carry out fructan metabolism in *A. tequilana*. The results also support the notion that as in the case of onion (*A. cepa*) enzymes with specific 1FFT activity may not be necessary in *Agave* species although this possibility needs to be confirmed by analysis of activity *in vitro* and/or in a heterologous system. Sequence alignments, conserved patterns of amino acids and differential expression patterns all support the classification of the different isoforms identified, although evidence from transcriptome data suggests that other isoforms still remain undetermined. The release of genomic sequences for *Agave* species will permit the definitive determination of numbers of isoforms for each enzyme and the analysis of gene regulatory elements. Conserved patterns of amino acids within the hypervariable loop may be a useful tool for initial identification and annotation of new sequences showing homology to members of PGHF32. Based on the observed expression patterns and the presence of fructan polymers, fructan metabolism must play an important role during flowering in these three Agave species and probably in most other species within the genus.

### Conflict of interest statement

The authors declare that the research was conducted in the absence of any commercial or financial relationships that could be construed as a potential conflict of interest.

## Abbreviations

PGHF32: plant glycoside hydrolase family 32
FEH: Fructan exohydrolase
1-SST: Sucrose:sucrose 1-fructosyltransferase
6G-FFT: Fructan:Fructan 6G Fructosyltransferase
6SFT: sucrose:fructan 6-fructosyltransferase
FFT: Fructan:fructan 1-fructosyltransferase
Vinv: Vacuolar invertase
Cwinv: Cell wall invertase.
