# Supplementary material for: New insights into plant glycoside hydrolase family 32 in Agave species
Source: Front Plant Sci. 2015 Aug 5;6:594. doi: 10.3389/fpls.2015.00594 (PMC4524927; doi:10.3389/fpls.2015.00594)
Supplement: Supplementary file 1 [file Table1.DOC]

Supplementary Table 1. List of accession numbers for all sequences used in alignments

| **Class** | **No. Accession** | **Species** | **Code** |
| --- | --- | --- | --- |
| Monocotyledon | EU981914 | Aegilops searsii | Asea1FFT |
| Monocotyledon | EU981909 | Aegilops searsii | Asea6SFT |
| Monocotyledon | FJ501983 | Aegilops tauschii | Atau1FFT |
| Monocotyledon | EU981910 | Aegilops tauschii | Atau6SFT |
| Monocotyledon | KD612321 | Aegilops tauschii | Atau6FEH |
| Monocotyledon | AF211253 | Agropyron cristatum | Ac6SFT |
| Monocotyledon | AJ006066 | Allium cepa | Ac1SST |
| Monocotyledon | Y07838 | Allium cepa | Ac6GFFT |
| Monocotyledon | AJ006067 | Allium cepa | AcInv |
| Monocotyledon | AB084283 | Asparagus officinalis | Ao6GFFT |
| Monocotyledon | AB195641 | Asparagus officinalis | Ao6FEH |
| Monocotyledon | GAHT01004229 | Agave deserti | Ad1SST-1 |
| Monocotyledon | GAHT01002436 | Agave deserti | Ad6GFFT-1 |
| Monocotyledon | GAHT01067452 | Agave deserti | AdVinv-1 |
| Monocotyledon | GAHT01048558 | Agave deserti | AdVinv-2 |
| Monocotyledon | GAHT01010554 | Agave deserti | AdVinv-3 |
| Monocotyledon | GAHT01069563 | Agave deserti | AdInv1 |
| Monocotyledon | GAHT01052073 | Agave deserti | AdInv2 |
| Monocotyledon | GAHT01012568 | Agave deserti | AdInv3 |
| Monocotyledon | GAHT01045893 | Agave deserti | AdCwin-1 |
| Monocotyledon | GAHT01020684 | Agave deserti | AdCwin-2 |
| Monocotyledon | GAHT01007433 | Agave deserti | AdCwin-3 |
| Monocotyledon | GAHT01044953 | Agave deserti | AdCwin-4 |
| Monocotyledon | GAHT01012621 | Agave deserti | AdFEH-1 |
| Monocotyledon | GAHT01008313 | Agave deserti | AdFEH-2 |
| Monocotyledon | EU026119 | Agave tequilana | Atq 1FFT |
| Monocotyledon | JN790053 | Agave tequilana | Atq1SST-1 |
| Monocotyledon | JN790054 | Agave tequilana | Atq1SST-2 |
| Monocotyledon | JN790055 | Agave tequilana | Atq6GFFT-1 |
| Monocotyledon | JN790056 | Agave tequilana | Atq6GFFT-2 |
| Monocotyledon | JN790057 | Agave tequilana | AtqCwinv-1 |
| Monocotyledon | JN790058 | Agave tequilana | AtqVinv-1 |
| Monocotyledon | GAHU01007017 | Agave tequilana | AtqVinv-2 |
| Monocotyledon | GAHU01060462 | Agave tequilana | AtqInv1 |
| Monocotyledon | KR138450 | Agave tequilana | AtqInv2 |
| Monocotyledon | GAHU01053508 | Agave tequilana | AtqCwin-2 |
| Monocotyledon | KR138451 | Agave tequilana | AtqCwin-3 |
| Monocotyledon | KR138454 | Agave tequilana | AtqFEH-1 |
| Monocotyledon | GAHU01085730 | Agave tequilana | AtqFEH-2 |
| Monocotyledon | KR138455 | Agave tequilana | AtqFEH-3 |
| Monocotyledon | GAHU01012712 | Agave tequilana | AtqFEH-4 |
| Monocotyledon | KR138448 | Agave striata | AstVinv-1 |
| Monocotyledon | KR138449 | Agave striata | AstVinv-2 |
| Monocotyledon | KR138452 | Agave striata | AstCwin-1 |
| Monocotyledon | KR138456 | Agave striata | AstFEH-1 |
| Monocotyledon | KR138447 | Agave victoriae-reginae | Avr1SST-1 |
| Monocotyledon | KR138453 | Agave victoriae-reginae | AvrCwin-1 |
| Monocotyledon | KR138457 | Agave victoriae-reginae | AvrFEH-1 |
| Monocotyledon | KR138458 | Agave victoriae-reginae | AvrFEH-2 |
| Monocotyledon | XM_003565097 | Brachypodium distachyoun | Bd6FEH |
| Monocotyledon | GQ247882 | Bromus pictus | Bp1FEH |
| Monocotyledon | FJ424612 | Bromus pictus | Bp6SFT |
| Monocotyledon | AJ297369 | Festuca arundinacea | Fa1SST |
| Monocotyledon | AJ605333 | Hordeum vulgare | Hv1FEH |
| Monocotyledon | AK354338 | Hordeum vulgare | Hv1FFT |
| Monocotyledon | X83233 | Hordeum vulgare | Hv6SFT |
| Monocotyledon | AJ534447 | Hordeum vulgare | HvCwinv |
| Monocotyledon | AK356300 | Hordeum vulgare | Hv6FEH |
| Monocotyledon | AJ623275 | Hordeum vulgare | HvInv |
| Monocotyledon | DQ016297 | Lolium perenne | Lp1FEH1 |
| Monocotyledon | AY245431 | Lolium perenne | Lp1SST |
| Monocotyledon | AF492836 | Lolium perenne | Lp6GFFT |
| Monocotyledon | DQ073969 | Lolium perenne | LpCwinv |
| Monocotyledon | DQ073968 | Lolium perenne | Lp6FEH |
| Monocotyledon | AF494041 | Lolium perenne | Lp6SFT |
| Monocotyledon | AAO21213 | Musa acuminata | MaCwinv |
| Monocotyledon | AB583555 | Phleum pratense | Pp6FEH |
| Monocotyledon | AB436697 | Phleum pratense | Pp6SFT |
| Monocotyledon | GU228513 | Poa pratensis | Ppr6SFT |
| Monocotyledon | AF276703 | Oryza sativa | OsInv2 |
| Monocotyledon | AF276704 | Oryza sativa | OsInv3 |
| Monocotyledon | AY578159 | Oryza sativa | OsCwinv2 |
| Monocotyledon | NM_001052039 | Oryza sativa | Os6FEH |
| Monocotyledon | AJ508387 | Triticum aestivum | Ta1FEH |
| Monocotyledon | AB088409 | Triticum aestivum | Ta1FFT1 |
| Monocotyledon | AB088410 | Triticum aestivum | Ta1FFT2 |
| Monocotyledon | AB029888 | Triticum aestivum | Ta1SST |
| Monocotyledon | AM075205 | Triticum aestivum | Ta6FEH |
| Monocotyledon | AB029887 | Triticum aestivum | Ta6SFT |
| Monocotyledon | AB089269 | Triticum aestivum | Ta61FEH |
| Monocotyledon | AJ516025 | Triticum aestivum | Ta1FEHw1 |
| Monocotyledon | AB089271 | Triticum aestivum | Ta6KEHw1 |
| Monocotyledon | AB089270 | Triticum aestivum | Ta6KEHw2 |
| Monocotyledon | AJ564996 | Triticum aestivum | Ta1FEHw3 |
| Monocotyledon | AJ635225 | Triticum aestivum | TaInv2 |
| Dicotyledon | X74514 | Arabidopsis thaliana | AtCwinv1 |
| Dicotyledon | AY039610 | Arabidopsis thaliana | AtInv1 |
| Dicotyledon | AB029310 | Arabidopsis thaliana | AtCwinv3 |
| Dicotyledon | AB479464 | Arctium lappa | Al1FFT |
| Dicotyledon | AB611034 | Arctium lappa | Al1FEH |
| Dicotyledon | AJ508534 | Beta vulgaris | Bv6FEH |
| Dicotyledon | AJ242538 | Cichorium intybus | Ci1FEHI |
| Dicotyledon | AY323935 | Cichorium intybus | Ci1FEHIIa |
| Dicotyledon | AJ295033 | Cichorium intybus | Ci1FEHa |
| Dicotyledon | AJ295034 | Cichorium intybus | Ci1FEHb |
| Dicotyledon | U84398 | Cichorium intybus | Ci1FFT |
| Dicotyledon | AJ509808 | Campanula rapunculoides | Cr1FEH |
| Dicotyledon | AJ000481 | Cynara scolymus | Cs1FFT |
| Dicotyledon | Y09662 | Cynara scolymus | Cs1SST |
| Dicotyledon | X75352 | Daucus carota | DcInv |
| Dicotyledon | AJ009756 | Helianthus tuberosus | Ht1FFT |
| Dicotyledon | AJ009757 | Helianthus tuberosus | Ht1SST |
| Dicotyledon | AAD01606 | Ipomoea batatas | IbInv1 |
| Dicotyledon | EU293871 | Lactuca sativa | Ls1FFT |
| Dicotyledon | AM231149 | Vernonia herbacea | Vh1FEH |
| Dicotyledon | AJ811625 | Viguiera discolor | Vd1FFT |
