# Supplementary figures and images for: New insights into plant glycoside hydrolase family 32 in Agave species
Source: Front Plant Sci. 2015 Aug 5;6:594. doi: 10.3389/fpls.2015.00594 (PMC4524927; doi:10.3389/fpls.2015.00594)

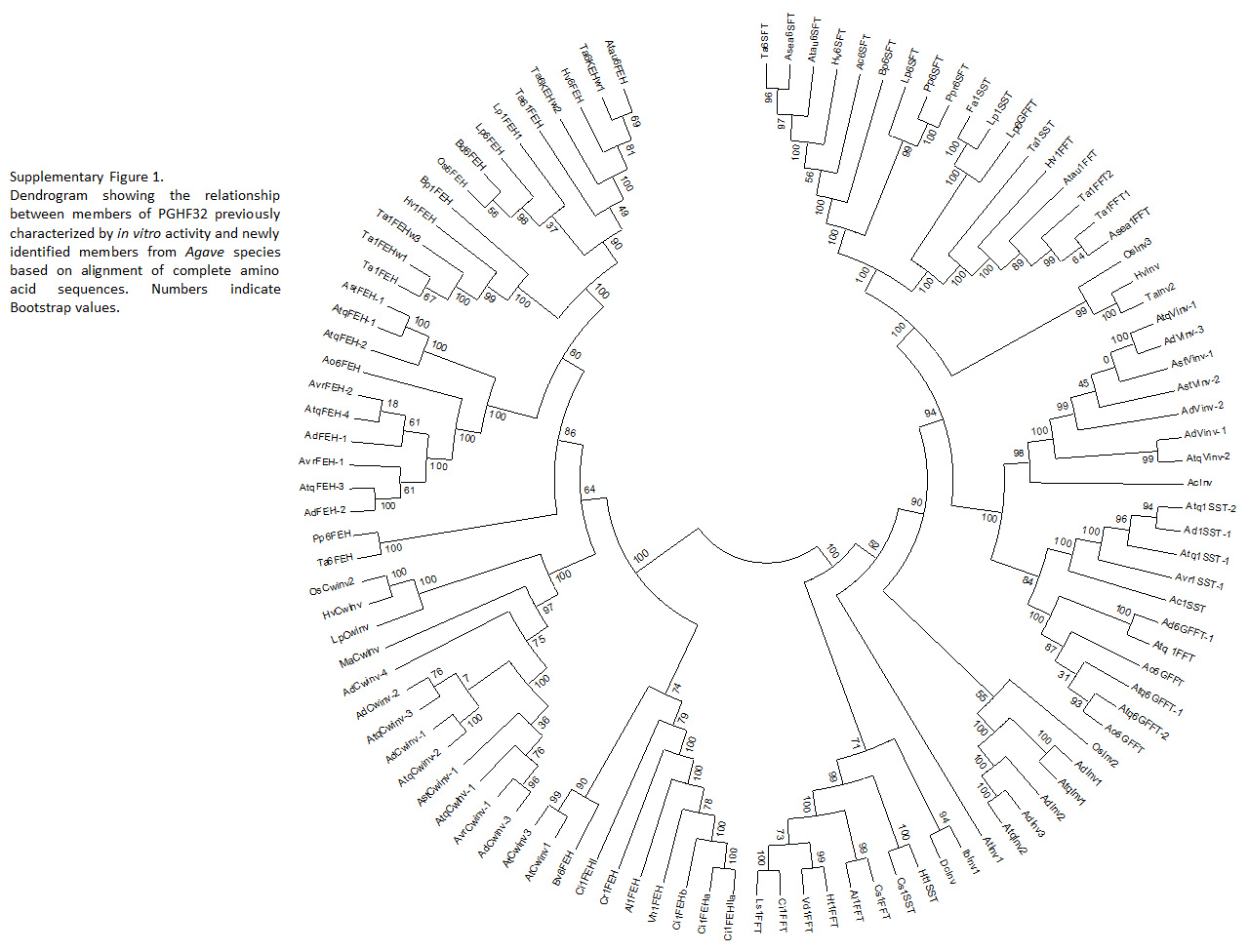

Supplement: Supplementary file 2 [file Image1.TIF]

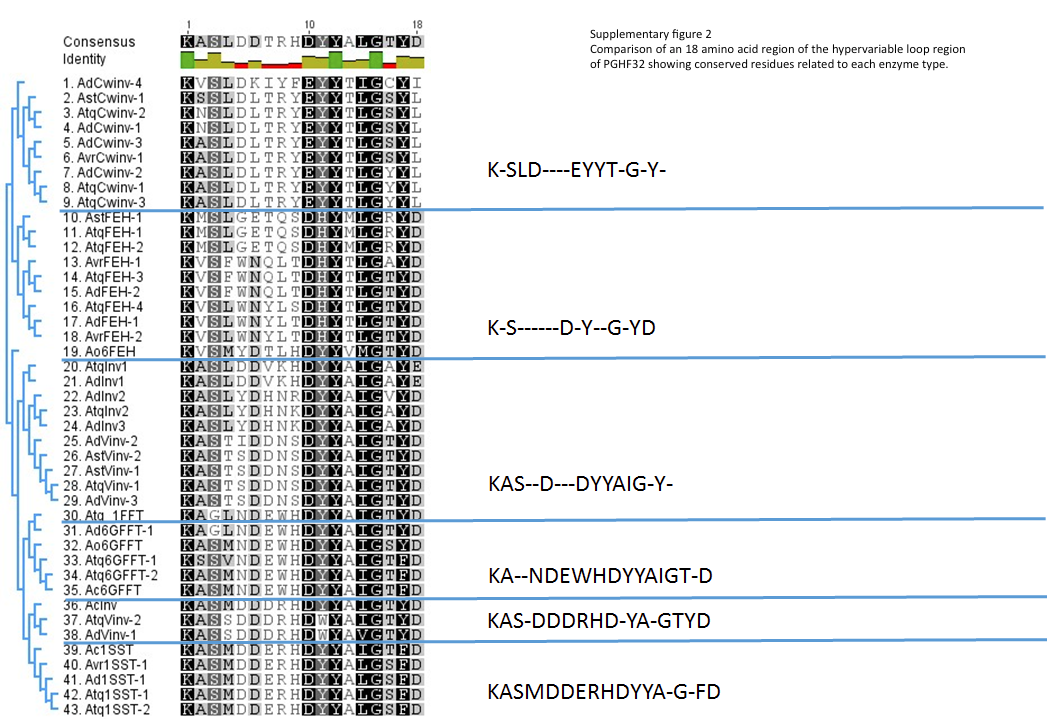

Supplement: Supplementary file 3 [file Image2.TIF]

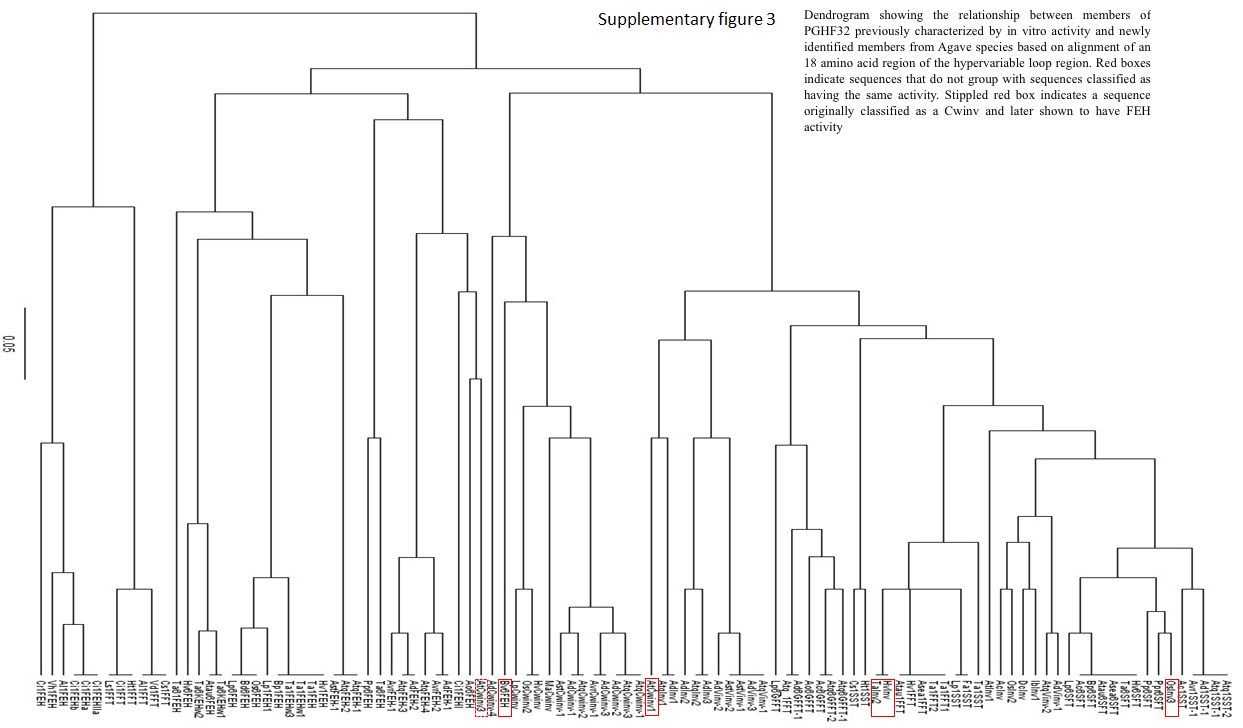

Supplement: Supplementary file 4 [file Image3.TIF]

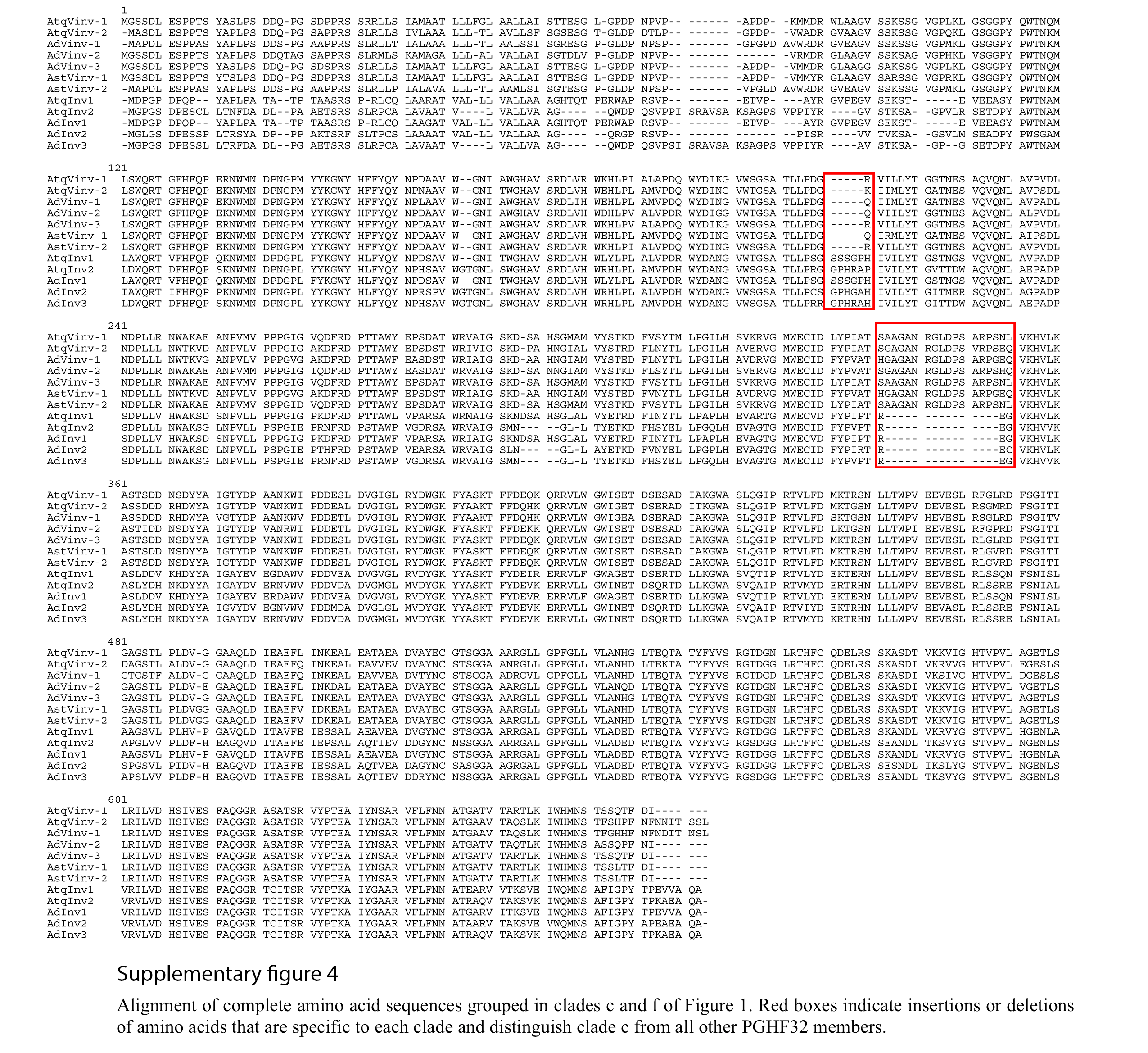

Supplement: Supplementary file 5 [file Image4.TIF]

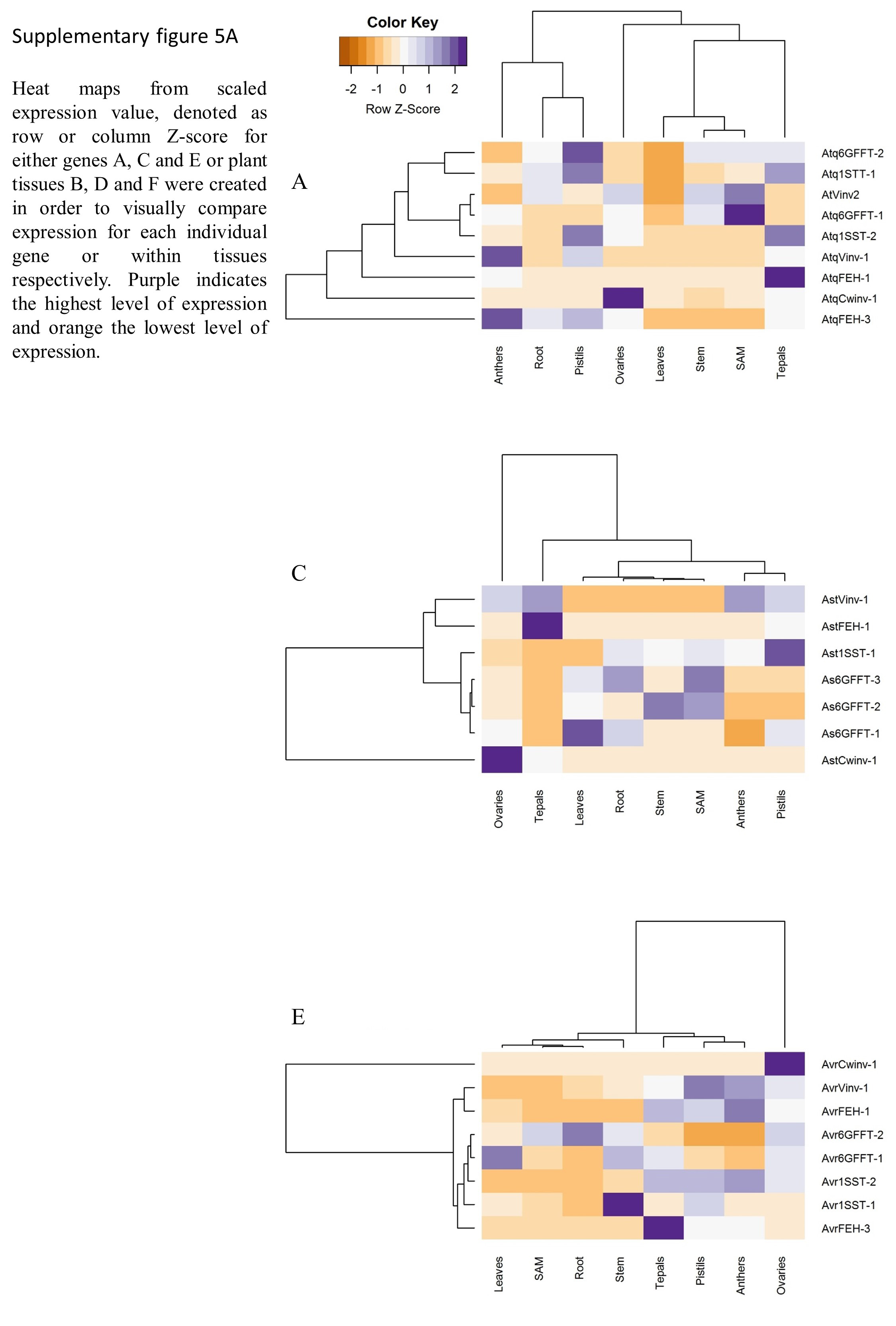

Supplement: Supplementary file 6 [file Image5.JPEG]

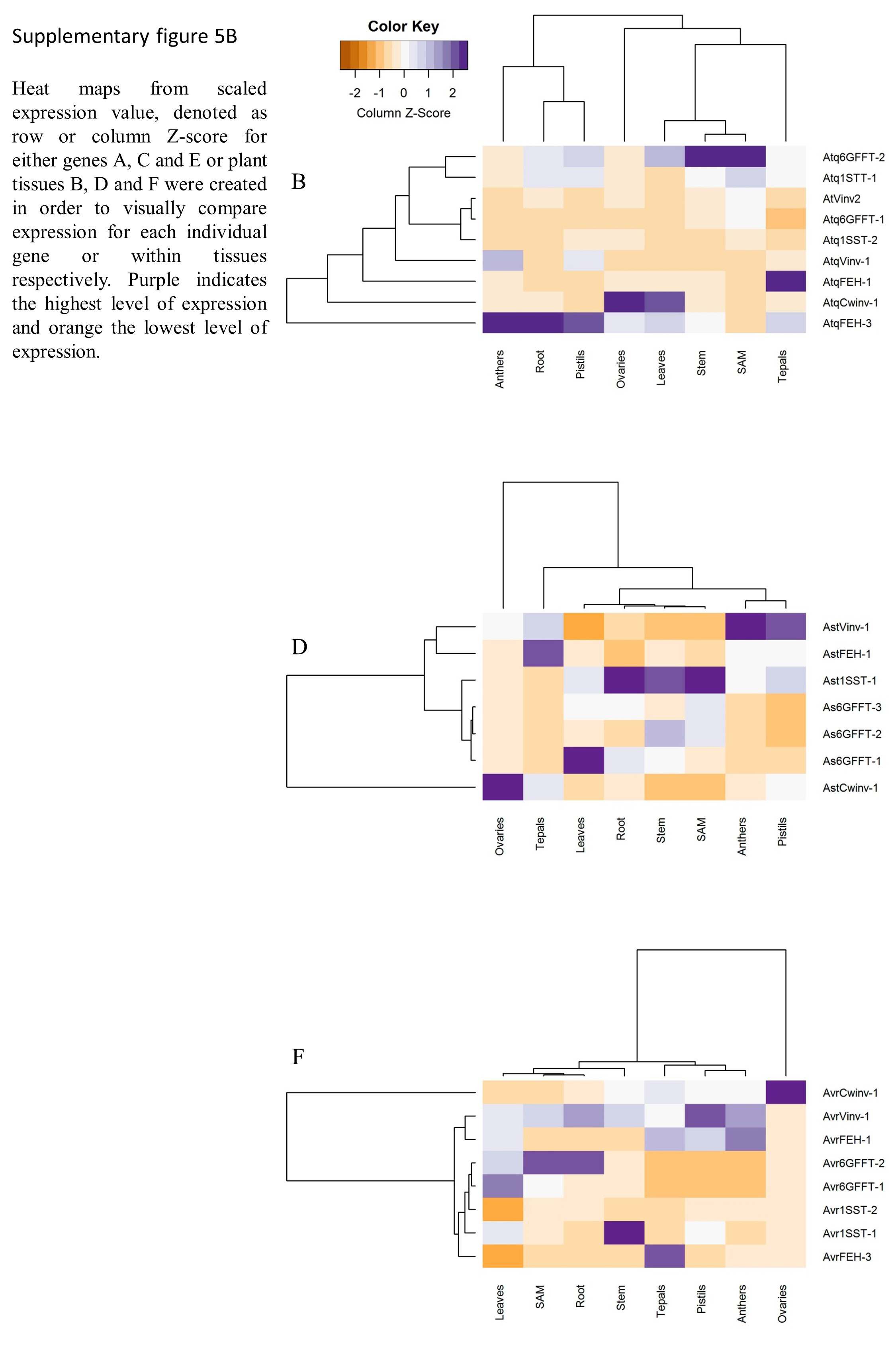

Supplement: Supplementary file 7 [file Image6.JPEG]
